# Osteometric Study of the Dorsal (Lister’s) Tubercle of the Radius in Relation to the Neighboring Anatomical Elements: Suprastyloid, Accessory, and Oblique Crests

**DOI:** 10.3390/life15020273

**Published:** 2025-02-11

**Authors:** Laura Octavia Grigoriță, Cătălin Prodan Bărbulescu, Norberth-Istvan Varga, Andreea Grigoriță, Monica Adriana Vaida, Loredana Gabriela Stana, Adelina Maria Jianu

**Affiliations:** 1 Department I—Discipline of Anatomy and Embryology, Faculty of Medicine, “Victor Babeş” University of Medicine and Pharmacy Timisoara, 2nd Eftimie Murgu Square, 300041 Timisoara, Romania; grigorita.laura@umft.ro (L.O.G.); vaida.monica@umft.ro (M.A.V.); loredana.stana@umft.ro (L.G.S.); adelina.jianu@umft.ro (A.M.J.); 2 2nd Surgery Clinic, “Pius Brinzeu” Clinical Emergency County Hospital, 300723 Timisoara, Romania; 3 Doctoral School, “Victor Babeş” University of Medicine and Pharmacy Timisoara, Eftimie Murgu Square 2, 300041 Timisoara, Romania; norberth.varga@umft.ro; 4 Faculty of Medicine, “Victor Babeş” University of Medicine and Pharmacy Timisoara, 300041 Timisoara, Romania; andreea171994@yahoo.com

**Keywords:** dorsal radial tubercle (Lister), oblique crest, suprastyloid crest, accessory crest, distal radial epiphysis, radial length

## Abstract

**Background:** The radius, a crucial bone in the human forearm, supports and facilitates complex movements like pronation and supination. Its anatomical landmarks, including Lister’s tubercle, provide vital attachment points for muscles, tendons, and ligaments involved in upper limb mobility. This study provides a detailed osteometric analysis of the dorsal radial tubercle of the radius, aiming to improve our understanding of its anatomy and clinical significance. **Material and Methods:** The study was conducted in the Department of Anatomy and Embryology, using 56 radius bones from cadavers. After applying inclusion and exclusion criteria, 46 bones remained in the study group. **Results:** The study found a significant positive correlation between the length of the radius and the width of the distal epiphysis. The distance from the suprastyloid crest to the dorsal radial tubercle (SC-DT) and the distal epiphysis width were strongly associated with the development of the distal radial epiphysis. The distance between the dorsal radial tubercle and the oblique ridge (OR-RI) and between the oblique ridge and the radial incisure (DT-OR) also showed a strong positive correlation with the distal epiphysis width. **Conclusions:** In conclusion, the osteometric study performed reveals significant correlations between the bony elements of distal radius epiphysis that can provide valuable information regarding anatomic variability and surgical treatment of distal radial epiphyseal fractures.

## 1. Introduction

### 1.1. Anatomy of the Radius—Osteology

The radius is an essential bone in the human forearm, playing a fundamental role in both structurally supporting and facilitating complex movements within the radio-carpal joint, such as pronation and supination [1,2]. The two bones of the forearm (radius and ulna) allow a wide range of forearm and wrist movements, which are essential for performing daily activities.

Nowadays, the anatomical perspective is becoming more and more broad, so that the distal radial epiphysis cannot be regarded as a static element. The importance of the radius extends beyond its mechanical function; its anatomical landmarks are vital attachment points for muscles, tendons and ligaments involved in forearm and hand mobility [3,4,5,6].

The “eye-catching” element that serves as the object of this study is Lister’s dorsal radial tubercle (*Tuberculum dorsale radii*) [7]. It is located at the dorsal aspect of the distal radial epiphysis distalis and serves as a pulley for the extensor pollicis longus (EPL) tendon, guiding its direction and facilitating thumb extension. In addition, it provides fixation points for the extensor retinaculum and serves as a passageway for a number of tendons, notably the abductor pollicis longus. [1,8]. In terms of its dimensions, Lister’s tubercle exhibits considerable variation in both height and length. Its height ranges from 2 to 6 mm, while its length ranges from 6 to 26 mm [9,10].

The anatomical landscape surrounding Lister’s tubercle is highly complex, with several critical neighboring structures influencing the overall biomechanics and functionality of the wrist and hand. Among these, the suprastyloid crest (*Crista suprastyloidea*), accessory ridge, and oblique ridge hold particular importance due to their proximity to the tubercle and their involvement in tendon and ligament attachments [5,8,9,11,12].

**Figure 1:**
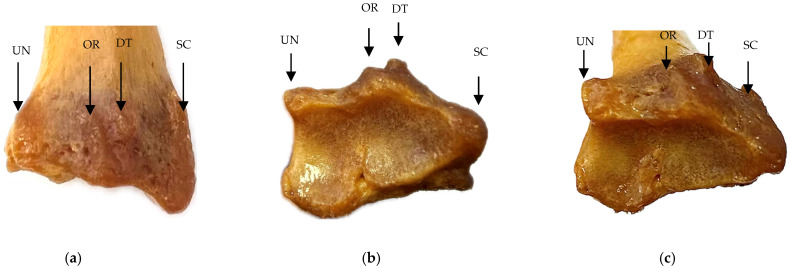
Anatomical elements of distal radial epiphysis: (**a**) anterior view; (**b**) inferior view; (**c**) antero-inferior view. UN—ulnar notch; OR—oblique ridge; DT—dorsal radial tubercle; SC—suprastyloid crest.

The suprastyloid crest, located proximal to Lister’s tubercle, exactly above the radial styloid process of radius, provides an attachment for the extensor retinaculum [11,13,14]. This ridge also plays a key role in stabilizing the tendons during wrist movements, especially in extension and supination. The accessory ridge, though less prominent, is a structural landmark that provides additional support for soft tissue elements, particularly tendons and ligaments involved in thumb and wrist motions, and is located between the suprastyloid crest and Lister’s tubercle. The oblique ridge, another important element of the distal epiphysis, plays an important role in stabilizing the distal radioulnar joint and is the insertion point for the ligamentous structures at this level. Specifically, this ridge facilitates rotational movement of the radius over the ulna during pronation and supination. The anatomical relationship between the oblique ridge and Lister’s tubercle is particularly important for understanding the mechanics of these movements and the distribution of forces on the wrist [2,15,16,17,18]. These anatomical elements and their relationships can be observed in Figure 1.

### 1.2. Anatomy of Radius—Myology

**Figure 2:**
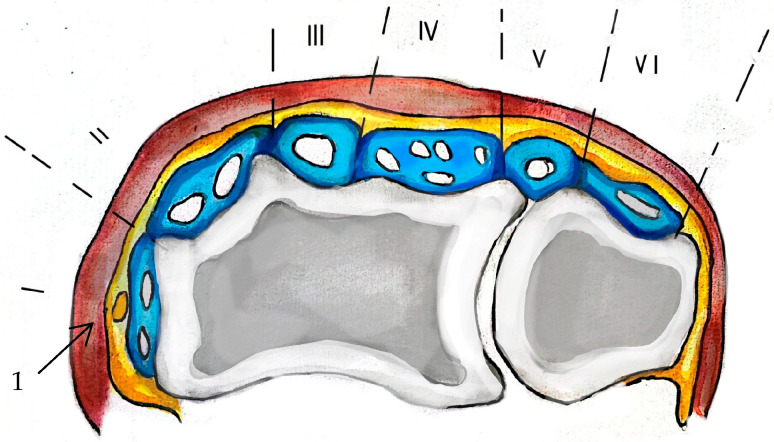
Distal radial epiphysis musculo-tendinous compartments. I. Abductor pollicis longus and extensor pollicis brevis. II. Extensor carpi radialis longus and brevis. III. Extensor pollicis longus. IV. Extensor digitorum communis and extensor indicis proprius. V. Extensor digiti minimi. VI. Extensor carpi ulnaris. 1. Superficial radial nerve.

Switching from osteology to myology, in the distal radial region on the dorsal aspect, the tendons of the extensor muscles of the forearm will pass under the extensor retinaculum at the wrist. The extensor retinaculum of the wrist (*Retinaculum extensorium carpi*) delimits six tendinous compartments by fibrous bands [15,19,20]. According to Figure 2, the musculo-tendinous compartments of the distal radial epiphysis, in order from radial to ulnar, are as follows:

First compartment (abductor pollicis longus, extensor pollicis brevis), second compartment (extensor carpi radialis longus, extensor carpi radialis brevis), third compartment (separated from compartment 2 by Lister’s tubercle; allows the passage of extensor pollicis longus), fourth compartment (extensor indicis, extensor digitorum), fifth compartment (extensor digiti minimi), sixth compartment (runs in the groove of the ulnar head and allows the passage of extensor carpi ulnaris) [15,19,21].

### 1.3. Aim of the Study

This study aims to bridge this gap by providing a comprehensive osteometric analysis of the dorsal radial tubercle of the radius. Through a detailed examination of its anatomical features, relationships with surrounding structures, and clinical significance, this research seeks to enhance the current understanding of the dorsal radial tubercle. Ultimately, these findings may contribute to improved diagnostic accuracy, the development of more effective surgical interventions, and better management of wrist and hand conditions involving this critical anatomical feature.

## 2. Materials and Methods

This study was designed as a retrospective observational analysis of dry bones conducted between 1 May 2024 and 1 September 2024. The study took place in Department I—Anatomy and Embryology Discipline of the “Victor Babes” University of Medicine and Pharmacy.

The research was conducted in the anatomical collection housed in the Department of Anatomy and Embryology. The bone specimens used in this study were extracted from cadavers and are part of the permanent collection of the department. During data acquisition, a total of 56 radius bones were collected.

These 56 radii came from bodies aged between 60 and 78 years, with a sex ratio of 39 female and 17 male bodies. In terms of ethnicity, the cadavers are of Romanian ethnicity.

The inclusion criteria were dry bones extracted from cadavers that showed no signs of fracture and were anatomically normal. Bones showing pathologic conditions, deformities or fractures were excluded from the study. Following the application of the inclusion criteria in the present study, 46 radius bones remained and 10 were excluded.

**Figure 3:**
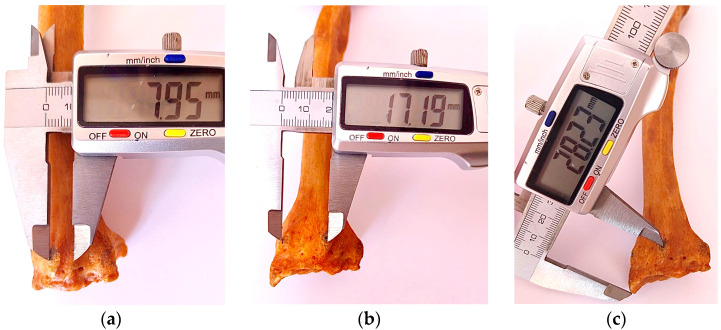
Osteometrical measurements of distal radius epiphysis. (**a**) distance (dorsal radial tubercle—oblique ridge); (**b**) distance (suprastyloid crest—dorsal radial tubercle); (**c**) distance (tip of radial radial styloid process—dorsal radial tubercle).

Technically, the measurements were carried out using a stainless steel gauge with a digital LED display produced by New Brand SUW-DIG. This device has a measuring range between 0 and 150 mm with an accuracy of ±0.03 mm. According to Figure 3, a series of specific measurements were taken from each radius bone using digital calipers with an accuracy of 0.01 mm. The following parameters were measured:

1. Radius length (radial head—apex of radial styloid process);
2. Radius length (lateral radial head—tip of radial styloid process) in millimeters;
3. Distance (suprastyloid crest—dorsal radial tubercle);
4. Distance (dorsal radial tubercle—oblique ridge);
5. Distance (oblique ridge—radial incision);
6. Width of radius (width of the distal epiphysis);
7. Distance (tip of radial styloid process—dorsal radial tubercle);
8. Presence/absence of accessory ridge;
9. Distance (suprastyloid crest—accessory ridge);
10. Distance (accessory ridge—dorsal radial tubercle).

Each measurement was recorded for both left and right radii.

Descriptive statistics were performed to calculate means and standard deviations for each measured parameter. A comparative analysis between left and right radii was conducted using paired *t*-tests for normally distributed data or non-parametric tests, such as the Wilcoxon signed-rank test, for non-normally distributed data. A *p*-value of less than 0.05 was considered statistically significant. All statistical analyses were conducted using SPSS software (version 29—September 2022).

## 3. Results

### 3.1. General Description of Radius Bone Samples

The osteometrical measurements of this study were performed on 46 radius bones, of which there were 18 right and 28 left radius bones.

The average length of the bones was 23.01 cm in the case of right radii, and 22.77 cm in the case of left radii. The average width of the distal epiphysis in the case of the right side was 33.29 cm, and 32.17 cm in the case of the left side. The maximum length of the right radii was 25.70 mm, and the maximum length of the left radii was 25.20 mm.

In the case of the right radius, the mean value of the SC-DT distance is 15.29 ± 2.88, compared to the same distance on the left side, which has a mean of 14.62 ± 1.91. There was a 2 cm wider epiphysis on the right side (33.28 ± 4.76) compared to 31.17 ± 3.48 on the left radius. All measurements are presented in Table 1.

### 3.2. Correlations Between Radial Measurements and Distal Epiphysis Width (Right Radius)

The Shapiro–Wilk test was used to assess the normality of distribution for the right radius data. The following variables were found to be normally distributed: length of the radius, distance between the suprastyloid crest and the dorsal radial tubercle, distance between the dorsal radial tubercle and the oblique ridge, distance between the oblique ridge and the radial incisure, and distal epiphysis width (*p* = 0.398, 0.793, 0.498, 0.183, and 0.647, respectively). As the assumption of normality was met, the suitability of Pearson’s correlation for analyzing the relationships between these anatomical features was confirmed.

As presented in Table 2, there is a statistically significant positive correlation between the length of the radius and the width of the distal epiphysis, so that in taller subjects, who clearly have a longer radius bone and wider distal epiphysis, the distances between the bone elements are longer, so the risk of developing tenosynovitis in the grooves crossed by tendons is smaller.

A very strong positive correlation was observed between the distance from the suprastyloid crest to the dorsal radial tubercle (SC-DT) and the distal epiphysis width (r = 0.900, *p* < 0.001). Similarly, the distance from the oblique ridge to the radial incisure (OR-RI) also showed a very strong positive correlation with distal epiphysis width (r = 0.879, *p* < 0.001). However, no significant correlation was found between the distance from the dorsal radial tubercle to the oblique ridge (DT-OR) and the distal epiphysis width (r = −0.063, *p* = 0.804).

These findings suggest that SC-DT and OR-RI are closely associated with the development of the distal radial epiphysis. Secondly, the strong correlation on the right side between the distance (SP-DT) and the width of the distal epiphysis can be quite clearly observed, with a value of r = 0.805 and a *p* < 0.01. In other words, the width of the distal epiphysis increases in direct proportion to the increase in the distance between the SP-DT. Another particular correlation is that between radius length and distance (SP-DT), which on the right side has an r = 0.629 and a *p* < 0.01.

This indicates a clear positive relationship; as the distances between the measured ridges increase, the transverse diameter of the distal epiphysis also increases.

### 3.3. Correlations Between Radial Measurements and Distal Epiphysis Width (Left Radius)

The following variables in the left radius data were found to be normally distributed: length of the radius, distance between the suprastyloid crest and the dorsal radial tubercle, distance between the dorsal radial tubercle and the oblique ridge, distance between the oblique ridge and the radial incisure, and distal epiphysis width (*p* = 0.084, 0.096, 0.395, 0.194, and 0.621, respectively). This confirms that Pearson’s correlation coefficient is suitable for analyzing the relationships between these anatomical features in the left radius sample.

Pearson correlation analysis was conducted to examine the relationships between the width of the distal radial epiphysis and other radial measurements in the left radius sample. The analysis revealed significant positive correlations between the distal epiphysis width and all the measured parameters. Specifically, the length of the radius showed a moderate positive correlation (r = 0.637, *p* < 0.001), while the distance between the suprastyloid crest and the dorsal radial tubercle demonstrated a strong positive correlation (r = 0.813, *p* < 0.001).

Furthermore, the distances between the dorsal radial tubercle and the oblique ridge (r = 0.522, *p* = 0.011) and between the oblique ridge and the radial incisure (r = 0.542, *p* = 0.003) also exhibited moderate positive correlations with the distal epiphysis width. These results indicate that larger values in the measured distances are generally associated with a wider distal radial epiphysis in the left radius bones. The values of the statistical results have been tabulated in Table 3.

Both the distance between the suprastyloid crest and the dorsal radial tubercle (SP-DT) and the length of the left radius show strong positive correlations with the distal epiphysis width (DEW). SP-DT exhibits a correlation coefficient of r = 0.765 (*p* < 0.01), while the length of the left radius shows a correlation coefficient of r = 0.711 (*p* < 0.01).

### 3.4. Accessory Crest

**Figure 4:**
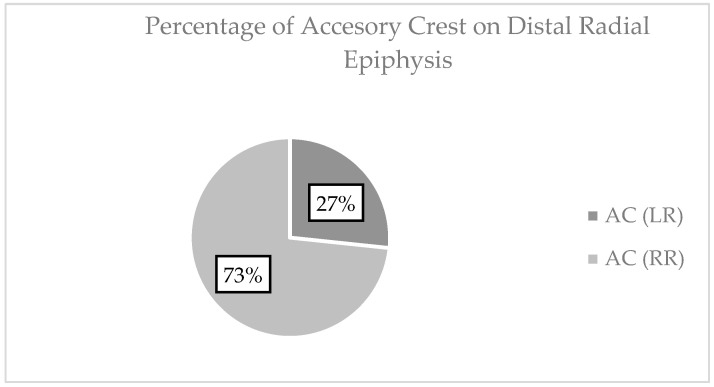
Distribution of accessory crest at the level of radius epiphysis.

According to Figure 4, on the left side, four of twenty-eight were detected. The presence of the accessory ridge correlates neither with the width of the distal epiphysis nor with the length of the radius. The r values are close to 0, *p* > 0.05. On the right side you have 11 of 18. It correlates neither with distal epiphysis width nor with radius length. The r values are close to 0, *p* > 0.05.

## 4. Discussion

### 4.1. Framing the Topic in the Specialized Literature

The distal radial epiphysis and its associated structures are critical for wrist biomechanics, particularly articulation with the scaphoid and lunate. Given the prevalence of traumatic injuries in this region, understanding these anatomical relationships is crucial for orthopedic and traumatological practice.

This study demonstrates a clear correlation between the distances between bony elements of the distal epiphysis and the epiphysis width. Greater distances between these ridges correspond to a larger transverse diameter of the distal epiphysis. Furthermore, a positive correlation exists between distal epiphysis width and radius length, suggesting that individuals with longer radii may have more spacious tendon compartments, potentially reducing the risk of compressive and inflammatory conditions.

An interesting observation is the asymmetry in the prevalence of the accessory ridge, an inconsistent anatomical element found more frequently on the right radius (73%) compared to the left (27%). This raises the question of whether its presence is related to hand dominance, as the majority of individuals are right-hand-dominant (approximately 90%). This observed asymmetry may reflect a form–function correlation, with increased osteogenic development on the dominant side.

Finally, while a moderate correlation between radius length and distal epiphysis width was observed bilaterally, the correlation was slightly stronger on the left side (r = 0.71) than the right (r = 0.637). This difference may be influenced by variations in the length of the radial radial styloid process.

Previous research has identified variations in the position of Lister’s dorsal radial tubercle. Agir et al. (2014) observed that the tubercle was positioned closer to the radial radial styloid process in eleven cases and closer to the ulnar notch in nine cases within their sample [22]. Clement et al. (2008) conducted measurements on 100 cadaveric specimens, reporting a range of 6–26 mm in tubercle length and 2–6 mm in height. They emphasized the clinical significance of the extensor pollicis longus (EPL) groove depth and tubercle height, particularly for volar plating procedures in distal radius fractures [9].

Chan et al. proposed a classification system for anatomical variants of Lister’s tubercle based on 360 MRI scans. Their system categorizes tubercle morphology into three types based on the relative heights of the radial and ulnar tips. This classification aids in identifying at-risk and rare variants, contributing to a more nuanced understanding of this structure.

### 4.2. Importance and Clinical Use of Measured Diameters

The dorsal radial tubercle’s clinical significance extends beyond anatomical variation. Pathological conditions, such as fractures, tendinitis, and degenerative changes, can significantly impair wrist function. Furthermore, the tubercle serves as a crucial landmark in surgical interventions, particularly for volar locking plate placement [6,23].

In concrete terms, measuring the diameters of the distal radial epiphyseal epiphysis and identifying anatomical variations serves two main directions.

#### 4.2.1. Anatomical, Educational and Research Perspectives

Specifically, here we refer to the use of these data in the teaching curriculum to help students appreciate interindividual variability in bone size and shape.

A second element is comparative left–right study, which is intended to help students understand the phenomenon of diversity of anatomical elements within the same organism.

The third argument is related to the fact that the measurements in this study can be reproduced in the practical laboratory, through live demonstrations, helping the student to learn to perform these measurements individually and thus to “see” these differences from one individual to another.

Another means of using these data, and the last in this enumeration, is to integrate and correlate them with imaging investigations, which could help students to correlate osteometry with imagistic studies, thus creating a “bridge” between preclinical and clinical disciplines [24,25].

#### 4.2.2. “Purely” Clinical Use of These Data

If we refer to the clinical use of these measurements—and it is imperative to do so—then these diameters can be used for the following: bone fracture risk assessment, orthopedic or ortho-plastic surgical planning, design of osteosynthesis material based on the measured biomodel, assessment of skeletal maturity, monitoring of arthritis and joint diseases and creation of three-dimensional models to replace bone by locating these diameters [26,27,28].

The first sub-point, bone fracture risk assessment, involves measuring the length and width of the bones, especially the distal epiphysis, to assess bone strength and fracture risk in that bone segment. Even more specifically, a wider epiphysis on a certain part of the bone, which represents a difference in an individual’s bone geometry, can be correlated with fracture susceptibility [8,9,29].

It has been mentioned about the assessment of skeletal maturity; here, we clearly refer to the measurement of these diameters, especially in children and adolescents, to estimate the age of skeletal maturity or even an incompletely closed growth plate, which again could influence a decision to implement a therapy [30].

Preoperatively, understanding the average radius dimensions is part of the preoperative planning for fracture fixation and for the perfect/optimal choice of implants.

The creation of three-dimensional bone bio-models based on these measurements could represent a futuristic technique for bone substitutes as an alternative to current osteosynthesis materials [31]. Specifically, one could use a segment of the distal radial epiphysis created in three-dimensional form based on these diameters, preoperatively measured via radioimagistic methods, which is probably more faithful than an osteosynthesis material, as it can more accurately mimic the bone configuration, making it a practical clone of the bone [31].

While previous studies have explored the tubercle’s role in fracture management and implant design, recent literature highlights the need for more comprehensive osteometric analyses, including its relationships with surrounding soft tissues [32]. Advances in minimally invasive techniques and 3D imaging have facilitated more detailed anatomical mapping, but a comprehensive understanding remains elusive. This study contributes to addressing this gap by providing a detailed osteometric analysis of the dorsal radial tubercle and its correlation with the distal radial epiphysis, furthering our understanding of this clinically relevant anatomical region.

With the increasing use of imaging-guided interventions and personalized surgical approaches, a thorough understanding of the dorsal radial tubercle’s anatomy is clinically essential. Recent efforts have focused on developing preoperative planning models that incorporate specific anatomical data, allowing surgeons to tailor interventions more effectively and minimize postoperative complications [10]. However, despite these advancements, the osteometric parameters of the dorsal radial tubercle remain incompletely defined, and their clinical significance remains under-explored. This study contributes valuable data to this area by providing a detailed osteometric analysis of the dorsal radial tubercle, including its dimensions and relationships with the surrounding skeletal structures.

### 4.3. Interindividual Anatomical Variability

As we can observe in the present study, the distal epiphysis of the radius shows marked interindividual anatomic variability, with major impact in orthopedic surgery, plastic surgery, trauma management, and anatomic studies.

Specifically, the variations of the distal radial epiphyseal epiphysis include variations in shape and dimensions, variations in bone density, variations in volar and dorsal inclination, and sex- and age-dependent variations. Summarized, all these “leave their mark” on surgical planning, on the management of bone fractures involving this segment, and last but not least, on the design of prosthetic materials in three-dimensional form.

Relating prosthesis or implant designs to the general anatomy of individuals represents a step forward in the field of orthopedics, which ensures the surgical success of patients. Relating the prosthesis or implant models to the specific anatomy of patient x ensures “ten steps forward” in terms of surgical success of the related intervention.

### 4.4. Limitations of the Current Study

This study acknowledges several limitations. First, the unequal number of right and left radius bones may have introduced bias into bilateral comparisons. While this discrepancy reflects the availability of specimens, future studies with balanced sample sizes are needed to confirm these findings. Second, the absence of imaging correlations, particularly MRI, limits the ability to comprehensively assess the relationship between bony landmarks and surrounding soft tissues. Although ethical concerns and technical challenges posed barriers to obtaining MRI data from cadaveric specimens, future research incorporating imaging techniques would enhance our understanding of this anatomical region. Finally, the relatively small sample size may limit the generalizability of the findings. While this study provides valuable insights into the osteometric characteristics of the dorsal radial tubercle, further research with larger samples is necessary to confirm these observations and extend their applicability to a broader population.

## 5. Conclusions

The present study, serving as a bridge in the anatomo-surgical landscape, reveals a series of correlations between the distances between these bony elements located at the distal radial epiphysis, highlighting the possibility of using these correlations in the surgical treatment of fractures at this level.

As a result of the interrelationship between these distances and the passage of the tendon elements between them, the bone elements that have been studied and that show statistically significant correlations—such as DT-OR and DEW, DT-SC and DEW, DT-SC and DEW, DT-SC and DEW, DT-SP and RL, and the presence or absence of the accessory ridge—provide useful information from two anatomical perspectives, which expresses the interindividual anatomical variability of the distal radial epiphysis in purely osteologic matters as well as surgical, showing the applicability of this variability in surgical planning and in actual surgical intervention.

Specifically, from a pedagogical point of view, the present measurements serve to open the horizon of medical students in terms of interindividual variability in bone size and shape, empowering them to perform measurements in the laboratory to familiarize themselves with osteometric research methods and integrate data with imaging investigations to bridge preclinical and clinical disciplines, thus improving their understanding of osteometry.

From a clinical and applied point of view, measured bone diameters and their correlations serve for bone fracture risk assessment, ortho-plastic surgery planning, design of osteosynthesis materials, assessment of skeletal maturity, arthritis monitoring and the creation of three-dimensional models for bone replacement. All of these should fit into the “bouquet” of pretherapeutic/pre-operative measures to prepare the orthopedic patient.

The whole framework created by this study represents only a small pillar regarding this area of research, a pillar that tries to earn a place in the perspective of teaching methods and another place in the perspective of management of an orthopedic patient.

## Figures and Tables

**Table 1:** General characteristics of radius bone samples.

Variable	Right Radius			Left Radius		
	Mean (±Std. Dev)	Min	Max	Mean (±Std. Dev)	Minimum	Maximum
RL	23.01 ± 1.94	19.4	25.7	22.77 ± 1.72	19.8	25.20
D(SC-DT)	15.29 ± 2.88	10.37	21.46	14.62 ± 1.91	11.48	18.93
D(DT-OR)	6.01 ± 0.66	4.95	7.59	5.60 ± 0.94	4.16	7.95
D(OR-RI)	11.98 ± 2.51	7.26	15.26	12.9 ± 2.3	7.31	18.50
D(SP-DT)	23.45 ± 2.70	19.76	30.50	21.9 ± 2.86	17	28
WDE	33.28 ± 4.76	24.92	41.97	32.17 ± 3.48	23.36	39.21

RL—radius length; D(SC-DT)—distance between suprastyloid crest and dorsal radial tubercle of Lister; D(DT-OR)—distance between dorsal radial tubercle of Lister and oblique ridge; D(OR-RI)—distance between oblique ridge and radial incisure; D(SP-DT)—distance between styloid process and dorsal radial tubercle of Lister; WDE—width of distal epiphysis of radius.

**Table 2:** Correlations of right radius diameters.

Variable	Correlation with DEW	*p*-Value
RL	0.711	<0.01
SC-DT	0.900	<0.01
DT-OR	−0.063	0.804
OR-RI	0.879	<0.01
SP-DT	0.805	0.000

RL—radius length; D(SC-DT)—distance between suprastyloid crest and dorsal radial tubercle of Lister; D(DT-OR)—distance between dorsal radial tubercle of Lister and oblique ridge; D(OR-RI)—distance between oblique ridge and radial incisure; D(SP-DT)—distance between styloid process and dorsal radial tubercle of Lister; WDE—width of distal epiphysis of radius.

**Table 3:** Correlations of left radius diameters.

Variable	Correlation with DEW (r)	*p*-Value
RL	0.637	0.000
SC-DT	0.813	0.000
DT-OR	0.522	0.011
OR-RI	0.542	0.003
SP-DT	0.765	0.000

RL—radius length; D(SC-DT)—distance between suprastyloid crest and dorsal radial tubercle of Lister; D(DT-OR)—distance between dorsal radial tubercle of Lister and oblique ridge; D(OR-RI)—distance between oblique ridge and radial incisure; D(SP-DT)—distance between styloid process and dorsal radial tubercle of Lister; WDE—width of distal epiphysis of radius.

## Data Availability

The original contributions presented in this study are included in the article. Further inquiries can be directed to the corresponding author.
